# Two Undervalued Functions of the Golgi Apparatus: Removal of Excess Ca^2+^ and Biosynthesis of Farnesol-Like Sesquiterpenoids, Possibly as Ca^2+^-Pump Agonists and Membrane “Fluidizers–Plasticizers”

**DOI:** 10.3389/fphys.2020.542879

**Published:** 2020-10-15

**Authors:** Arnold De Loof, Liliane Schoofs

**Affiliations:** Research Group of Functional Genomics and Proteomics, Department of Biology, KU Leuven, Leuven, Belgium

**Keywords:** Golgicrine activity, juvenile hormone, SERCA, SPCA, thapsigargin, corpus allatum, hormone receptor, calcitox

## Abstract

The extensive literature dealing with the Golgi system emphasizes its role in protein secretion and modification, usually without specifying from which evolutionary ancient cell physiological necessity such secretion originated. Neither does it specify which functional requirements the secreted proteins must meet. From a reinterpretation of some classical and recent data gained mainly, but not exclusively, from (insect) endocrinology, the view emerged that the likely primordial function of the rough endoplasmic reticulum (RER)–Golgi complex in all eukaryotes was not the secretion of any type of protein but the removal of toxic excess Ca^2+^ from the cytoplasm. Such activity requires the concurrent secretion of large amounts of Ca^2+^-carrying/transporting proteins acting as a micro-conveyor belt system inside the RER–Golgi. Thus, (fitness increasing) protein secretion is subordinate to Ca^2+^ removal. Milk with its high content of protein and Ca^2+^ (60–90 mM vs. 100 nM in unstimulated mammary gland cells) is an extreme example. The sarco(endo)plasmatic reticulum Ca^2+^-ATPases (SERCAs) and SPCA1a Ca^2+^/Mn^2+^ transport ATPases are major players in Ca^2+^ removal through the Golgi. Both are blocked by the sesquiterpenoid thapsigargin. This strengthens the hypothesis (2014) that endogenous farnesol-like sesquiterpenoids (FLSs) may act as the long sought for but still unidentified *agonist*(s) for Ca^2+^-pumps in both the ER and Golgi. A second putative function also emerges. The fusion of both the incoming and outgoing transport vesicles, respectively, at the *cis*- and *trans*- side of Golgi stacks, with the membrane system requiring high flexibility and fast self-closing of the involved membranes. These properties may—possibly partially—be controlled by endogenous hydrophobic membrane “fluidizers” for which FLSs are prime candidates. A recent reexamination of unexplained classical data suggests that they are likely synthesized by the Golgi itself. This game-changing hypothesis is endorsed by several arguments and data, some of which date from 1964, that the insect *corpus allatum* (CA), which is the major production site of farnesol-esters, has active Golgi systems. Thus, in addition to secreting FLS, in particular juvenile hormone(s), it also secretes a protein(s) or peptide(s) with thus far unknown function. This paper suggests answers to various open questions in cell physiology and general endocrinology.

## Introduction

**FIGURE 1:**
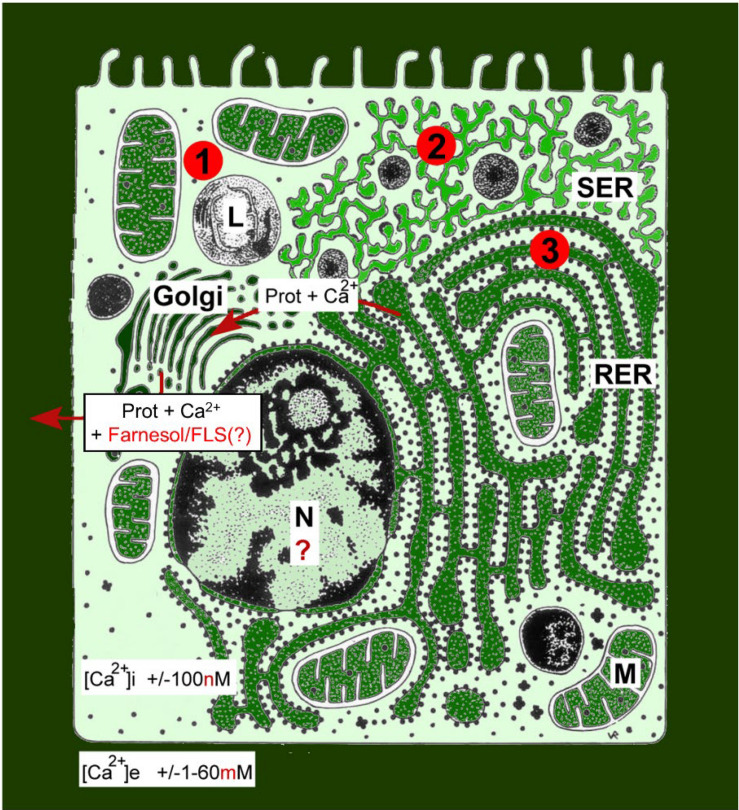
Schematic representation of the Ca^2+^ gradients (adapted from De Loof, 2017a). The different shades of green are not meant to give an exact representation of differences in Ca^2+^ concentration. L, lysosome; N, nucleus; M, mitochondrion; RER, rough endoplasmic reticulum; SER, smooth endoplasmic reticulum. By just looking at the ultrastructure of cells and by evaluating how abundant the SER and RER are, one can make plausible guesses about the gross outline of their Ca^2+^ homeostasis system, as well as of some of its non-genomic effects on those enzyme systems that are involved in lipid, steroid, and protein syntheses. This is due to the fact that numerous enzymes, the activity of which is (partially) controlled by the Ca^2+^ gradient over their membrane, are anchored in these SER and RER membrane systems. The red dots with 1, 2, and 3 correspond to the mechanisms 1–3 for keeping [Ca^2+^]i low (see text). Copyright permission: Own work (De Loof, 2017a), licensed under Creative Commons license.

In cell- and organismal animal physiology, Ca^2+^ is best known for its beneficial effects, e.g., in the construction of calcareous skeletons in various animals, as a secondary messenger in signaling pathways, in neuronal activity, in muscle contraction, etc. It is less known that Ca^2+^ is the most abundant toxin on earth, and that exactly this toxicity—at increasing cytoplasmic concentrations to above 100 nM—forms the basis for the cited beneficial effects. The huge Ca^2+^ concentration gradients over the plasma membrane, ranging from an average of about 1–2 mM extracellularly vs. 100 nM in the cytoplasm of unstimulated cells, in combination with the fact that the plasma membrane is not fully impermeable to Ca^2+^ (mainly due to Ca^2+^ channels and pumps), result in a constant drive for Ca^2+^ to enter the cell. Figure 1 gives a visual impression of how Ca^2+^ is gradient-wise distributed not only over the plasma membrane, but intracellularly as well. It illustrates the challenges a eukaryotic cell faces due to the fact that it has to maintain a low concentration of free intracellular Ca^2+^ in an environment in which the extracellular Ca^2+^ concentration is at least 20,000 times higher.

When for whatever reason(s) excess Ca^2+^ enters the cell, this excess has to be removed as quickly as possible. Small amounts of Ca^2+^ can be removed by plasma membrane ATPases and functionally related enzymes (Mechanism 1 in Figure 1) and by temporary storing excess Ca^2+^ in the lumina of intracellular membrane systems, in particular the ER and the mitochondria (Mechanism 2 in Figure 1; Orrenius et al., 2003; Groenendyk et al., 2004). When these mechanisms do not suffice, a third system is mobilized, namely, removal of Ca^2+^ through the rough endoplasmic reticulum (RER)–Golgi system (Mechanism 3 in Figure 1). The secretion of Ca^2+^ along with milk proteins from mammary gland cells is an extreme example (see section “‘From Physiological Necessity’: Which Role For Ca^2+^ Homeostasis?”). If even this mechanism fails, the Ca^2+^ overload will induce cell death/apoptosis (Nicotera and Orrenius, 1998; Orrenius et al., 2003). Selective massive (programmed) cell death that is physiologically and developmentally controlled occurs in, e.g., the gut epithelium, during complete metamorphosis of insects, and in humans in the later stages of Alzheimer’s disease (De Loof and Schoofs, 2019a).

## Always Keep in Mind That There Is No “Goal” in Evolution, Only “Physiological Necessity”

Our counterintuitive proposal that protein secretion by the RER–Golgi is subordinate to its role in removal of excess Ca^2+^ is likely to meet with initial skepticism. At first sight, the reason for skepticism seems very logical and understandable, but it is nonetheless erroneous. The “milk example” illustrates why. If people are asked why female mammals after having given birth start producing milk, the almost unanimous answer is: “To feed and provide immune protection to their young!” But if one next asks if the female deliberately *plans* to engage in milk production, hesitation emerges. This hesitation turns into negation upon asking: “Do the mammary gland cells ‘know’ that they have to produce milk because a young/baby is waiting to be fed?” Of course not. But why is galactopoiesis initiated at all, if there is no “goal” at all to do so? The right answer is that females start secreting milk because of hormonally controlled *physiological necessity*. The “milk case” illustrates that there is no “goal” in evolution (De Loof, 2015, 2017b), perhaps some exceptions not taken into account (Pookottil, 2013), and that the ways followed to increase fitness can be very ingenious.

## “From Physiological Necessity”: Which Role for Ca^2+^ Homeostasis?

The drop in progesterone and estrogen titers prior to birth giving, combined with increased release of some brain hormones such as prolactin, which facilitates the entry of Ca^2+^ into the mammary gland cells, and oxytocin, which is instrumental to milk ejection, is causal to this “necessity.” Secondarily, the beneficial effect of milk, with its high concentrations of proteins, Ca^2+^, lipids, etc., increases the fitness of the lactating female and the population to which she belongs. Lactation got conserved in evolution because it increases fitness.

The “milk case” raises several cell physiological issues. One concerns the high concentration of Ca^2+^ in milk, up to 60–100 mM (Wuytack et al., 2003). This concentration is about 600,000 to 1 million times higher than the free Ca^2+^ concentration in the cytoplasm of unstimulated cells. Again, the usual answer to the question why milk is so extremely rich in Ca^2+^ is: “Because the developing baby/young needs lots of Ca^2+^, among other things, for the construction of its calcareous endoskeleton.” And next: “Do the mammary gland cells add so much Ca^2+^ to the milk fluid with this “intention?” Of course not. They do so to get rid of the massive amounts that enter the gland cells resulting from the increase in permeability to Ca^2+^, which is caused by the “lactation-promoting hormones” produced by the brain of lactating females. The ultimate reason for secreting Ca^2+^ is that excess Ca^2+^ is very toxic (section “Basic Mechanisms in Ca^2+^ Homeostasis”) and has to be eliminated as quickly as possible. The suckling young survives being fed with a potentially toxic nutrient because he or she neutralizes the excess incoming Ca^2+^ by uploading it in deposits of Ca^2+^ that are no longer toxic, in particular in the developing skeleton and in intracellular Ca^2+^ stores, such as the lumina/cisternae of the endoplasmic reticulum, the mitochondria, and Golgi. The mammary gland cells do not succumb under the high Ca^2+^ concentrations because they make use of the conveyor belt of transport proteins produced in the RER and next secreted by the Golgi, packed into vesicles.

## Basic Mechanisms in Ca^2+^ Homeostasis

**FIGURE 2:**
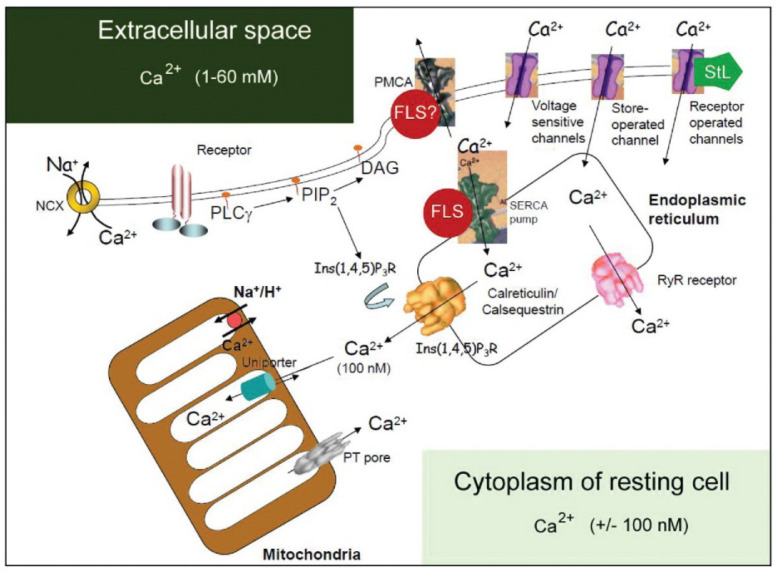
Classical view of the major players in the system that regulates intracellular Ca^2+^ compartmentalization. Cellular Ca^2+^ import through the plasma membrane occurs largely by receptor-operated (e.g., glutamate receptors), voltage-sensitive and store-operated channels. Once inside the cell, Ca^2+^ can either interact with Ca^2+^-binding proteins or become sequestered into the endoplasmic reticulum (ER) or mitochondria. The largest Ca^2+^ store in cells is found in the ER or sarcoplasmic reticulum, with local Ca^2+^ concentrations reaching millimolar levels. Ca^2+^ levels in the ER are affected by the relative distribution of sarco(endo)plasmatic reticulum Ca^2+^-ATPase (SERCA) pumps and of inositol-1, 4, 5-triphosphate [Ins(1, 4, 5)P_3_] receptors [Ins(1, 4, 5)P_3_Rs] and ryanodine receptors (RYRs), as well as by the relative abundance of Ca^2+^-binding proteins (calreticulin, calsequestrin) in the ER or sarcoplasmic reticulum. The cytosolic Ca^2+^ concentration in unstimulated cells is kept at approximately 100 nM by both uptake into the ER and Ca^2+^ extrusion into the extracellular space by the plasma membrane Ca^2+^-ATPase (PMCA). ER Ca^2+^ release is triggered by agonist stimulation through the generation of Ins(1, 4, 5)P_3_ through hydrolysis of phosphatidylinositol-4,5-biphosphate [PtdIns(4,5)P_2_] operated by a phospholipase C (PLCγ). The mitochondria take up Ca^2+^ electrophoretically through a uniport transporter and can release it again through three different pathways: reversal of the uniporter, Na^+^/H^+^-dependent Ca^2+^ exchange, or as a consequence of permeability transition pore (PTP) opening. The PTP can also flicker to release small amounts of Ca^2+^. Ca^2+^ efflux from cells is regulated primarily by the PMCA, which binds calmodulin and has a high affinity for Ca^2+^. Ca^2+^ efflux might also be mediated by the Na^+^/Ca^2+^ exchanger (NCX). [Ca^2+^], calcium concentration; DAG, diacylglyceride. This figure was kindly provided by Prof. S. Orrenius. It served as the template for a figure in Orrenius et al. (2003). Regulation of cell death: the calcium-apoptosis link. Nature Rev. Cell Biol. 2003; 4:552–565; https://doi.org/10.1038/nrm1150. The figure was slightly modified by adding the red dots with farnesol-like sesquiterpenoid (FLS), suggesting a role for endogenous sesquiterpenoids (FLSs) as agonists for Ca^2+^-ATPases. Such role is more probable for sarco(endo)plasmatic reticulum Ca^2+^-ATPases (SERCAs) than for PMCAs (hence the question mark). In 2003, the role of the Golgi system in Ca^2+^ homeostasis was not yet well documented and therefore not represented in this figure. With copyright permission for both the figure and the legend from the publisher and from Prof. S. Orrenius. This modified figure was published before in De Loof (2017a), with copyright permission (Open Access).

If one is not familiar with the principles of Ca^2+^ homeostasis, the statement that Ca^2+^ is the most abundant toxic pollutant in the aqueous environment of cells but that it nonetheless became the best communicator may sound strange at first encounter. Calcium is an ambivalent messenger (Krebs et al., 2015; Carafoli and Krebs, 2016). On one hand, it is essential to the correct functioning of cell processes, but, if not carefully controlled spatially and temporally within cells, it generates various severe cell dysfunctions, and even cell death (Nicotera and Orrenius, 1998; Orrenius et al., 2003; Carafoli and Krebs, 2016). The very origin of the Ca^2+^ homeostasis system dates back to ancestral prokaryotes as a survival system preventing Ca^2+^-mediated cell damage (Case et al., 2007). It further developed at the unicellular stage of eukaryote evolution (Plattner and Verkhratsky, 2015, 2016). Later on, mechanisms of signaling became diversified, reflecting multiplication and specialization of Ca^2+^-regulated cellular activities (Figure 2). They have been very well conserved in evolution (Plattner and Verkhratsky, 2015). The toxicity of Ca^2+^ and the counterbalancing by endogenous farnesol-like sesquiterpenoids (FLSs) (De Loof et al., 2015; De Loof and Schoofs, 2019e) resemble the story of the evolution of O_2_ toxicity, the underlying free radical [reactive oxygen species (ROS)] mechanisms and the role of antioxidants (Zhu et al., 2016).

The Ca^2+^ homeostasis system with its numerous molecular players (Ca^2+^ pumps, Ca^2+^ channels, calmodulin, etc., Figure 2) produced a steep (see section “‘From Physiological Necessity’: Which Role For Ca^2+^ Homeostasis?”) concentration gradient between extracellular and intracellular compartments, which had both signaling function and survival importance. The latter is illustrated by the fact that even relatively (prolonged) moderate increases in cytosolic Ca^2+^ concentrations above 100 nM are incompatible with life. Thus, the most basic *activity of the Ca^2+^ homeostasis system is to keep cytosolic Ca^2+^ very low*, not higher than 100 nM. Remarkably, which mechanisms control this “keeping Ca^2+^ low” are only partially understood, in particular, the role of farnesol-like endogenous sesquiterpenoids remains undervalued until to date (De Loof and Schoofs, 2019e; this paper).

Rising concentrations of Ca^2+^ can alter the 3D conformation of some types of macromolecules, in particular, of proteins as well of chromatin/DNA. This forms the basis of the potential toxicity of Ca^2+^, as well as for the fact that Ca^2+^ can act as a secondary messenger in various signaling systems (De Loof, 2017a; De Loof and Schoofs, 2019b). Muscle contraction is an example of the effect of a sudden Ca^2+^ increase resulting from its release from the lumen of the smooth endoplasmic reticulum. Maintaining “livable gradients” of Ca^2+^ is a most important task for all eukaryotic cells. We next focus on some novel insights in the role of the Golgi system and of endogenous FLSs in achieving this goal.

### The Golgi Apparatus: A Complex System

**FIGURE 3:**
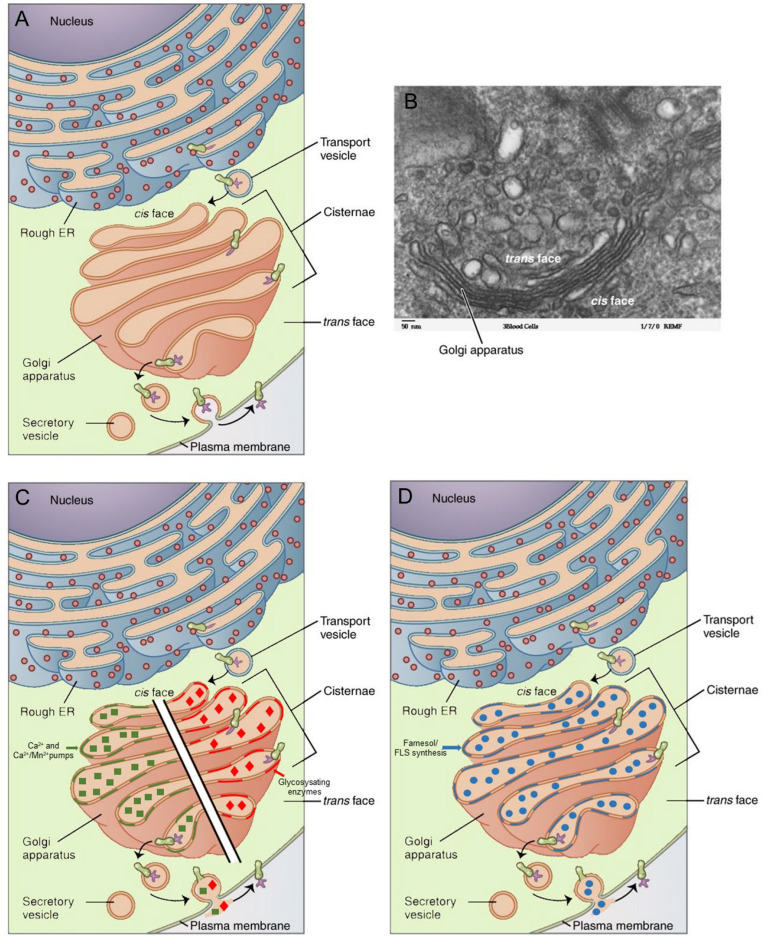
Golgi apparatus in context of the secretory pathway: **(A)** Schematic representation (Golgi in salmon pink). **(B)** Electron micrograph. **(C)** Schematic representation of the Ca^2+–^ and the Ca^2+^/Mn^2+^ pumps as well as of glycosylating enzymes in the membranes. **(D)** The Golgi membranes also contain enzymes for the synthesis of farnesol/farnesol-like sesquiterpenoid (FLS). The major function of the rough endoplasmic reticulum (RER)–Golgi apparatus is the removal of excess Ca^2+^ from the cytoplasm, an activity that requires the synthesis of cell type-specific transport proteins in the RER. From here, proteins are sent to the Golgi apparatus, which organizes, modifies (e.g., by glycosylation), packages, and tags them. Some of these products are transported to other areas of the cell, and some are exported from the cell through exocytosis. Enzymatic proteins are packaged as new lysosomes (or packaged and sent for fusion with existing lysosomes) (Nakano and Luini, 2010). A reanalysis of classical data from insect endocrinology (see text) revealed that the Golgi apparatus is the likely site of synthesis of farnesol and farnesol-like sesquiterpenoids (FLSs), the juvenile hormones of insects being the best known ones. Copyright permission: **(A–D)** All under Creative Commons license CC BY 4.0. **(A,B)** Original files unchanged from Wikipedia File 0314 Golgi Apparatus.jpg (Created May 18, 2016). **(C,D)** Slightly modified from this file.

According to Barlow et al. (2018), the Golgi’s characteristic morphology of multiple differentiated compartments organized into stacked flattened cisternae (Figure 3) fused together in a continuous ribbon structure (Gosavi and Gleeson, 2017) is one of the most recognizable features of modern eukaryotic cells. How it originated and how it is maintained are not well understood. It may reasonably be assumed that the composition and lateral organization of the Golgi membranes are not less complex than those of the plasma membrane (Jacobson et al., 2019). According to Barlow et al. (2018), golgins, which are omnipresent in eukaryotes, are prominent proteins implicated in Golgi structure, and this since the common eukaryotic ancestor. The Golgi apparatus has many functions (Makhoul et al., 2018). Its role in protein secretion, a process that usually includes protein modification, is best known. Golgi membranes harbor a set of glycosylating enzymes (Figure 3C) that attach various sugar monomers to proteins moving through the apparatus. Other functions include mitosis, DNA repair, stress responses, Ca^2+^ homeostasis (Zhang et al., 2020), lysosome production (Nakano and Luini, 2010), autophagy, apoptosis, inflammation, and prostaglandin synthesis (Smith and Malkowski, 2019). The Golgi apparatus also interacts with the microtubule and actin networks (Kulkarni-Gosavi et al., 2019). It is a central meeting point for the endocytotic and exocytotic systems in eukaryotic cells (Barlow et al., 2018). Last but not least, through logical deduction, De Loof and Schoofs (2019b, d) came to the conclusion that the Golgi system is the most probable site of synthesis of endogenousFLSs) (section “The Golgi Apparatus as a Probable Subcellular Site of Synthesis of Farnesol/Farnesol-Like Sesquiterpenoid: Origin of This Idea”).

### Pumping Excess Ca^2+^ Out of the Cell: The Golgi System as Part of the Ca^2+^ Homeostasis System

The best known Ca^2+^ pumping systems (active transporters) are the plasma membrane Ca^2+^-ATPases (PMCAs) and the sarco/endoplasmic-reticulum Ca^2+^-transport ATPases (SERCAs with various splice variants). Figure 2 illustrates the complexity of the system. In addition, animal cells also contain a less characterized P-type Ca^2+^-transport ATPase, PMR1/SPCA Ca^2+^/Mn^2+^-transport ATPase (Van Baelen et al., 2002), which is encoded by two genes that have various splice variants. This P-type Ca^2+^-transport ATPase is mainly targeted to the Golgi apparatus (Wuytack et al., 2003). It provides the Golgi with the Ca^2+^ and Mn^2+^ necessary for the production and processing of secretory proteins. An N-terminal Ca^2+^-binding motif in the Ca^2+^/Mn^2+^-transport ATPase SPCA1 regulates the secretory pathway in particular in cells with a high Ca^2+^ load (e.g., mammary gland cells during lactation) or in cells with a low ATP content such as keratinocytes (Chen et al., 2019). Phylogenetic analysis showed that SPCA1 may be older than the SERCA pump and related to a putative ancestral Ca^2+^ pump (Wuytack et al., 2003). The Golgi system can function as a Ca^2+^ store, which can be involved in setting up cytosolic Ca^2+^ oscillations (Wuytack et al., 2003).

## The Link Between Ca^2+^ Pumping by Sarco(Endo)Plasmatic Reticulum Ca^2+^-Atpases and Endogenous Sesquiterpenoids

### Fifty Years Ago: Arrest of Farnesol/Juvenile Hormone Production Impairs Golgicrine Secretory Activity in an Insect. Temporary Rescue Possible

By electron microscopy, De Loof and Lagasse (1970) discovered that the ultrastructure of fat body cells of the Colorado potato beetle (*Leptinotarsa decemlineata*) changes when the farnesol/juvenile hormone (JH) synthesizing glands, the *corpora allata* (CA), are inactivated by microsurgical removal. In addition, they also observed this phenotype when the beetles were raised in conditions with a short photoperiod (= less than 12-hour light per day). Other tissues were not investigated, and sites of JH synthesis other than the CA were not known at that time (see later).

The most drastic effect in the fat body cells was on the functioning of the Golgi systems: the secretion of vesicles was totally disturbed. The outgoing transport vesicles no longer fused with the plasma membrane but instead fused with one another, thereby forming large protein bodies (for figure, see De Loof and Schoofs, 2019b, or De Loof and Schoofs, 2019a). At that time, this effect was (erroneously: see sections “‘From Physiological Necessity’: Which Role For Ca^2+^ Homeostasis?” and “From Physiological Necessity”: Which Role For Ca^2+^ Homeostasis?) interpreted as being physiologically relevant and beneficial for depositing reserves in preparation of hibernation/diapause.

We now believe that the effect is in fact a step toward programmed cell death. However, at that time, the role of Ca^2+^ in the induction of apoptosis was not yet known. Orrenius et al. (2003) advanced it as a causal link about 30 years later. Ablation of the CA not only affects the Golgi systems but nearly all aspects of cell physiology, including lipid, protein, and ecdysteroid biosynthesis, the multiplication of mitochondria, etc. It visualized in a few images that farnesol esters are indeed the master hormone(s) in insect physiology and development (De Loof and Schoofs, 2019b, d). Both reimplantation of active CA and administration of synthetic JH (dissolved in an oil to act as a slow-release formula) temporarily (= within a few days) rescued the observed phenotype and thus demonstrated that *absence of farnesol/JH* is the real cause of the cell physiological effects induced by allatectomy (De Loof and Lagasse, 1970).

### Another “Spark” of Insight: The Sarco(endo)plasmatic Reticulum Ca^2+^-ATPase Pump Blocker Thapsigargin Is, Like Farnesol/Juvenile Hormones, a Sesquiterpenoid

Sarco(endo)plasmatic reticulum Ca^2+^-ATPases can interact with a variety of molecules. The number of interacting proteins keeps growing (Vandecaetsbeek et al., 2011). One of the well-known agents is thapsigargin, a potent blocker of both the SERCA pump (Rogers et al., 1995) and the SPCA1a Ca^2+^/Mn^2+^ transport ATPase present in the Golgi apparatus (Chen et al., 2017, 2019; Smaardijk et al., 2017). Thapsigargin is extracted from the plant *Thapsia garganica*, hence its name. Structurally, it is classified as a sesquiterpene lactone. By blocking the ability of the cell to pump calcium into the sarcoplasmic and endoplasmic reticula, thapsigargin raises the cytosolic (intracellular) Ca^2+^ concentration. Store depletion can secondarily activate plasma membrane calcium channels, allowing an influx of calcium into the cytosol. Depletion of ER calcium stores leads to ER stress and ultimately leads to cell death (Zhang et al., 2020).

The similarity of cell death induction in a wide variety of eukaryotes by administration of thapsigargin as well as by silencing the production of JH by the CA of insects with a complete metamorphosis (= Holometabola) triggered the search for the cause of this similarity. Is, perhaps, thapsigargin toxic because it displaces a natural sesquiterpenoid ligand from its binding site on Ca^2+^-pump(s) thereby blocking them (De Loof et al., 2014)? It is known since long that farnesol and its JH-esters (Röller et al., 1967; Röller and Dahm, 1968) are sesquiterpenoids, but for thapsigargin, this property is only rarely mentioned. Despite intensive research, the natural ligand for this binding still remains unknown. We think, without having experimental proof, that for engaging in Ca^2+^ pumping, SERCAs and other Ca^2+^ pumps need the presence of an endogenous FLS as an *agonist* of the pumps (De Loof et al., 2014; De Loof, 2017a).

### Some Ca^2+^ Channel Types Are Receptors for Farnesol

In addition to a suggested but not yet experimentally proven role of FLS as agonists of SERCAs and possible other Ca^2+^ pumps (De Loof, 2017a), direct experimental proof for a receptor role of farnesol for a voltage-gated Ca^2+^ channel has been described by the electrophysiologists Roullet et al. (1999) and Luft et al. (1999). For more details, see De Loof and Schoofs (2019a,b).

In combination, the picture emerges that farnesol/FLSs act on keeping intracellular Ca^2+^ low by concurrently inhibiting the influx of Ca^2+^ through (some types of) Ca^2+^ channels and acting as agonists of Ca^2+^ pumps that remove excess Ca^2+^. This made De Loof and Schoofs (2019b) state that the membrane receptor(s) of farnesol/JH is the integrated Ca^2+^ homeostasis system in its totality. Even ecdysteroids and hydrophobic vertebrate steroid hormones may act in a similar way.

### Nuclear Receptor(s) for Juvenile Hormones: Methoprene-Tolerant (Met), the Putative Insect Nuclear Juvenile Hormone Receptor, Remains Controversial

Yamamoto (1988) suggested, based on solid experimental results, that a membrane protein mediates an effect of JH that involves calcium and kinase C. Much later, Jindra et al. (2015a, b) advanced the view that the transcription factor Methoprene-tolerant is, in their opinion, the key/master receptor for JH: no need for a membrane receptor, Ca^2+^, and kinase C. This view gained a rather wide acceptance among molecular biologists, but not among cell biologists. The major objection is that, up to the present day, it has not been experimentally demonstrated that farnesol/JHs, which are highly hydrophobic molecules, ever enter the nucleus with its internal hydrophilic watery environment. As long as the *in situ* binding between JH and its Met-transcription factor inside the nucleus has not been experimentally proven, Met does not meet the required conditions to be classified as a “genuine receptor.” According to De Loof and Schoofs (2019a,d,e), there is no problem to classify MET as a JH *target*. Even more, it is probable that Met is a Ca^2+^-sensitive target (De Loof and Schoofs, 2019e, and this paper).

Farnesol/JH influences many other targets, most of them residing in membranes as cited before (section “The Link Between Ca^2+^ Pumping by Sarco(Endo)Plasmatic Reticulum Ca^2+^-Atpases And Endogenous Sesquiterpenoids”). To our knowledge, no data are available on nuclear receptors for farnesol in chordates. Wigglesworth (1969) suggested that the difference in biological activity of some 40 compounds he tested in a typical JH bioassay was quantitative rather than qualitative, and that such compounds first act at the plasma membrane, with secondary effect in the nucleus as a result. Wigglesworth (1969) also reported that JH analogs are not active in a watery environment or in the presence of wetting agents. This brings the hydrophobicity issue, which is very relevant for sesquiterpenoids and some steroid hormones, in focus (section “The Hydrophobicity Issue in Farnesol/Farnesol-Like Sesquiterpenoid Transport and Mode of Action. The “Waterway Mode” Versus the Lipid Membrane Way or “Inbrome Way””).

## A Still Continuing Search: How Many Sites of Farnesol/Farnesol-Like Sesquiterpenoid Synthesis Are in the Body?

### The Situation in Insects Is Best Documented

There are several reasons why more experimental data on the site of synthesis and the functions of farnesol have been advanced in insects and not in any chordate/vertebrate species. First, until recently (De Loof et al., 2015; De Loof and Schoofs, 2019e), farnesol was known neither as a hormone nor as an “inbrome” (De Loof et al., 2015) in vertebrates. Its only role in chordates was thought to be one of the intermediates in the mevalonate biosynthetic pathway that leads to the synthesis of squalene, and next to cholesterol and steroids, without having any function on its own. Logically, that should have changed when the electrophysiologists Roullet et al. (1999) and Luft et al. (1999) demonstrated that farnesol is a potent blocker of N-type voltage-gated Ca^2+^ channels in the brain of some rodents and of humans. It did not. Hence, the question where endogenous FLSs are synthesized never became a topic worth investigating in chordates. In contrast, in insects, it started with the pioneering work of Kopec (1922) about a century ago, with his discovery that the insect brain is an endocrine gland. Later, key pioneers were sir Vincent Wigglesworth with his discovery of JH synthesized by the CA, and Carroll M. Williams with the serendipitous discovery that the abdomen of male *Hyalophora cecropia* moths was a repository of high amounts of JH (references in Riddiford, 1994; Watt and Davey, 1996; Nijhout, 1998). It took many years before it became clear that the CA were not the only site of synthesis of farnesol/JHs. An indirect indication in favor of multiple sites of synthesis farnesol/JHs was advanced by Castillo-Gracia and Couillaud (1999). They found that the catalytic site of the enzyme farnesyl-diphosphate synthase, which plays a role in isoprenoid biosynthesis, was present in the brain, ovary, fat body, and CA samples (= all tissues with active Golgi systems), but not in muscle, a tissue that does not engage in substantial protein secretion. In the CA, the encoding gene for farnesyl-diphosphate synthase is overexpressed.

### Endocrine-, Exocrine-, and Golgicrine Farnesol/Juvenile Hormone Synthesis

The successive steps in this development have recently been reviewed by De Loof and Schoofs (2019d, e). In short, up to 1995, the CA were assumed to be the sole site of synthesis of JHs. That changed when Borovsky et al. (1994a, b) reported that, in addition to the CA, both male and female gonads of mosquitoes also synthesized JHs *de novo*. Another two decades later, Paroulek and Sláma (2014) refined this addition by showing that in the moth *H. cecropia*, the model insect from which JH I had been originally extracted and chemically identified, JH is synthesized *de novo* in the male accessory glands (= to some extent the counterpart of the mammalian prostate gland) and this in a developmental stage in which the CA are inactive. They named it “exocrine JH synthesis.” They also reported that this male accessory gland (MAG) JH was not secreted into the hemolymph, but instead left the male body along with secretory proteins that during copulation are transferred into the female’s genital system. This selectivity, or better unidirectionality, in JH secretion triggered De Loof and Schoofs (2019d) to search for an explanation for this at first sight unusual transport of a hydrophobic/lipophilic hormone. When upon analyzing Schmialek’s (1961) experimental data, the idea emerged that the most plausible and logical answer to the question how farnesol can end up in the lumen of the alimentary canal of larval mealworms, was that this requires a key contribution of Golgi systems of the gut’s epithelial cells. Indeed, the Golgi system with its membranes that harbor various enzymes including some P450 enzymes, a family of enzymes that plays important roles in both the biosynthesis and inactivation of 20-hydroxyecdysone and JHs (Iga and Kataoka, 2012), is the only cell organelle that can explain the unidirectional transport of JH along with secretory proteins in *Hyalophora*. This way, the idea of “Golgicrine farnesol/FLS synthesis and secretion” was born. Immediately, the question emerged whether such “Golgicrine activity” is compatible with JH synthesis by the CA.

### Gland Cells of the Corpora Allata Have Golgi Systems: A Problem Emerges

The first mention of the presence of active Golgi systems in the CA was published by Scharrer (1964) in the cockroach *Leucophaea maderae*. Similar results were later published by Bradley and Edwards (1979) in CA of the house cricket *Acheta domesticus*. Although it was known since long that Golgi systems serve a role in the secretion of proteins, the possibility that the “juvenilizing factor” secreted by the CA might be a protein was not considered at that time. The farnesol line of thinking (Schmialek, 1961) dominated the scene at that time. In fact, it continues to do so until today. Hence, the question: “What type of protein/peptide do the Golgi of the CA secrete?” still remains unanswered. It cannot be excluded that the key function of the CA is *not* the secretion of JH, but of an as yet unknown sort of proteinaceous/peptidic growth factor. In particular, in the recent decade, several analyses of the peptidome of the CA have been undertaken. Liu et al. (2012) could assign a total of 41 ion masses present in the CC–CA complex of fifth instar *Bombyx mori* but no clear-cut candidate for a peptide that is only secreted by the Golgi of the CA was found in this or other (e.g., Nouzova et al., 2018b) mass spectrometric studies.

One should remember that Sláma (2013, 2015) since long postulated the possibility that the “real JH” might be the protein/peptide secreted by the Golgi of the CA. Almost nobody listened. In our opinion, answering this question is of prime importance for avoiding that some researchers continue working along wrong premises and hypotheses.

Maybe a putative “Golgi-growth stimulating peptide” has not yet been found because it could be totally absent from early metamorphosis on up to adult emergence. In such situation, only indirect arguments can be advanced. Short Neuropeptide F might be a good candidate (arguments and references in De Loof and Schoofs, 2019b; Fadda et al., 2019). When sNPF peptide was injected into adult female locusts, the stimulation of ovarian development was so pronounced that Cerstiaens et al. (1999) thought that they discovered a potent growth hormone of insects or, alternatively, a releasing hormone for such growth hormone. Later research showed that it is a much more complex issue and that sNPF plays, among other functions, a prominent role in the control of feeding (Carlson et al., 2013; Fadda et al., 2019). In addition, changes in microRNAs may also play a role (Nouzova et al., 2018a).

## The Golgi Apparatus as a Probable Subcellular Site of Synthesis of Farnesol/Farnesol-Like Sesquiterpenoid: Origin of This Idea

Not only the question which protein/peptide is secreted by the Golgi of the CA, but also the questions where exactly farnesol/FLS is synthesized in the gland cells and how it leaves the CA cells remain unanswered and deserve innovative thinking.

### Peter Schmialek’s Identification of Farnesol and Farnesal in the Mealworm’s Excrements

Schmialek (1961) was the first to chemically characterize two compounds that were active in the *Tenebrio* assay for detecting JH activity, namely, the sesquiterpenoids farnesol and farnesal. They were not extracted from tissue or whole-body extracts but from excrements of the mealworm *Tenebrio molitor*. The question how these compounds could end up in the lumen of the alimentary canal was asked, but could not be answered. The hypothesis was that their transport was achieved through the Malpighian tubule system as degradation products of the true—but at that time still unidentified—JH. But the Malpighian tubule system does not transport such hydrophobic compounds. Upon reexamining Schmialek’s data, De Loof and Schoofs (2019d) logically deduced that the more probable explanation is that the Golgi system of the epithelial cells of the gut play a role in the unidirectional transport of endogenous sesquiterpenoids into the gut’s lumen.

### Paroulek and Sláma: In the Male Accessory Glands of *H. cecropia*, Exocrine Juvenile Hormone I Is *Unidirectionally* Transported

As already cited before, Paroulek and Sláma (2014) solved the problem of the site of synthesis of the high amounts of JH-active material in the MAGs of *H. cecropia.* It had been thought for many years that the MAGs only function as a repository for the JHs that were already synthesized in the CA (Shirk et al., 1976). However, the authors proved that not the CA but the MAGs themselves synthesize the JH they accumulate, and that they do not release it into the hemolymph. The consequence is that MAG JH I should not have been denominated as a “hormone.” Later, when it was shown that the CA also produce JH I, which they do release into the hemolymph, it rightly acquired the status of “hormone,” at least for JHs that end up in the hemolymph. An intriguing issue remained unanswered: How to explain that MAG-JH does not diffuse into the hemolymph, but that instead it is unidirectionally transported along with secretory proteins produced by the MAGs into the female genital system during copulation.

### The Hydrophobicity Issue in Farnesol/Farnesol-Like Sesquiterpenoid Transport and Mode of Action. The “Waterway Mode” Versus the Lipid Membrane Way or “Inbrome Way”

**FIGURE 4:**
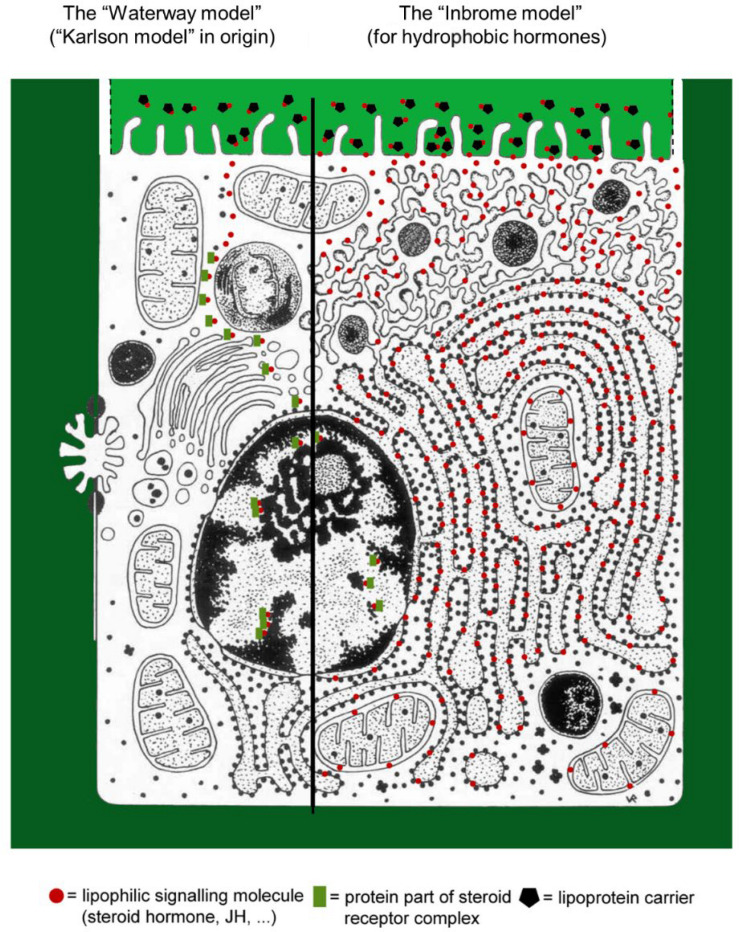
Two models for the entry and intracellular transport of small non-proteinaceous/peptidic hormones in cells. **(Left panel)** According to Karlson and Sekeris (1966) the ecdysteroid molting hormone of insects (20-hydroxy ecdysone or 20E), which is moderately water-soluble, could pass the plasma membrane by simple diffusion without eliciting any signaling (e.g., through Ca^2+^) at the level of this membrane. Next, it would diffuse through the cytoplasm and end up in the nucleus. Here, it would (in)activate specific genes. In the 1960s–1970s, this concept was expanded toward being also valid for lipohilic/hydrophobic molecules such as some vertebrate-type sex steroids and to insect juvenile hormone(s). Later, this model got complemented with cytoplasmic protein transporters for the transport of lipophilic/hydrophobic molecules through the cytoplasm into the nucleus. This model, despite justified criticisms, is still widely used to date. **(Right panel)** The “intramembrane transport model” or “inbrome model.” In particular for hydrophobic small hormones, e.g., the juvenile hormone esters of farnesol, the inbrome mode of action model was originally advanced by De Loof (2015). It says that such hydrophobic hormones are transported in the blood/hemolymph by a lipoprotein that delivers the hormone at the plasma membrane, in which the hormone will enter through hydrophobic interaction. Next, the hormone will start diffusing through the entire system of all connected membrane systems: plasma membrane, smooth endoplasmic reticulum (SER)–rough endoplasmic reticulum (RER), nuclear envelope, Golgi apparatus, mitochondria, etc. These lipid bilayer membranes are liquid and act as a solvent and transport system for hydrophobic molecules. During the diffusion, the hydrophobic molecules will encounter many proteins that are anchored in the membranes. They all have hydrophobic transmembrane stretches of amino acid chains. Some membrane proteins may act as genuine receptors. The interaction with actors of the Ca^2+^ homeostasis system is important. The lighter green color at the top of this figure does not mean that here the Ca^2+^ concentration is lower, but to make the details readable. Intracellular Ca^2+^ gradients (Figure 1) are not represented. Own work, modified after De Loof (2017a, Open Access): Copyright permission under Creative Commons License.

To date, the almost general consensus is that, after its synthesis somewhere in the gland cells of the CA, JH freely diffuses through the cytoplasm toward the plasma membrane of the gland cells, next inserts itself into its lipid bilayer, and next “jumps” to lipoproteins circulating in the hemolymph. This view neglects the consequences of a most important chemical property of farnesol/JHs, namely, that it is only very moderately soluble in water. Thus, without a hydrophobic carrier, it cannot freely diffuse through a watery/hydrophilic environment. Recent textbooks (e.g., Raven et al., 2019) pay attention to the hydrophobicity issue. The “exocrine JH” denomination of MAG-JH by Paroulek and Sláma (2014) suggests another way of transport of JH. After its synthesis in the Golgi system itself, JH gets attached to hydrophobic parts of (transport) proteins that are on their way from the RER to the cell’s exterior—to end up in the hemolymph. Apparently, this hydrophobic interaction is sufficiently strong to prevent the diffusion of JH through the membranes of the Golgi system. In such scenario, exocrine JH would also have an endocrine function, which is not the case of the MAGs of *Hyalophora*, because MAG-JH does not end up in and is not transported by the hemolymph. Thus, by logical deduction, the conclusion can be reached that farnesol/FLS, after their synthesis in membranes of the Golgi (not yet experimentally proven!), distributes itself between the membranes of the Golgi and hydrophobic stretches of amino acids present in the Golgi’s lumen where they can be further modified. Because both the membrane and the content of the lumen of the Golgi move toward the *trans* side where pinching off happens, farnesol/FLS also move along. We propose the term “lipid membrane way mode of transport” or “inbrome way of transport” (De Loof et al., 2015) of small hydrophobic molecules for such transport (Figure 4). In fact, the membrane system of cells acts as a liquid hydrophobic solvent system through which hydrophobic hormones and other solutes can freely diffuse. This oil canal system complements the “the waterway mode” for the transport of, e.g., water-soluble hormones: through the blood or through the hydrophilic cytoplasm.

## Function(s) of Farnesol/Farnesol-Like Sesquiterpenoids in the Golgi Membranes

### Sarco(endo)plasmatic Reticulum Ca^2+^-ATPase and SPCA1a Ca^2+^/Mn^2+^ Transport ATPase Have a Binding Site for Some Sesquiterpenoids

We only elaborate on the plant toxin thapsigargin because it is a sesquiterpenoid that, in our opinion, likely acts by displacing a natural endogenous sesquiterpenoid ligand(s) from its binding site on the SERCA pump. That ligand could be farnesol itself or a farnesol-like sesquiterpenoid (FLS) (De Loof, 2017a). As already mentioned in section “Another “Spark” of Insight: The Sarco(endo)plasmatic Reticulum Ca^2+^-ATPase Pump Blocker Thapsigargin Is, Like Farnesol/Juvenile Hormones, a Sesquiterpenoid,” both the SERCAs and the Ca^2+^/Mn^2+^ Ca^2+^-transport ATPases have a binding site for the plant toxin thapsigargin, which blocks these pumps. This toxin is a sesquiterpene lactone. It binds to SERCA in a hydrophobic funnel-like cavity open to the cytosol formed by TM3, TM5, and TM7 transmembrane loops of the SERCA pump and prevents the movement of the helices relative to each other. Nine out of the 10 transmembrane loops of SERCA undergo rearrangements during a pumping cycle, enabling the release of Ca^2+^ into the lumen of the ER and Golgi and, on the cytoplasmic side, to create a pathway for entry of new Ca^2+^ ions (for references, see De Loof, 2017a). To date (2020), experimental validation of this hypothesis or the identification of other putative endogenous ligands is still missing. The fact that the Golgi apparatus seems to be a major site of synthesis of farnesol/FLS strengthens the hypothesis that such compounds may be, one way or another, agonists of the Ca^2+^ pumps that play prominent roles in the RER–Golgi secretory pathway. Because thapsigargin acts as a SERCA blocker in various eukaryotes and because SERCAs are very well conserved in evolution, it can be assumed that the binding is well conserved as well. However, this awaits experimental validation.

### Facilitating Membrane Self-Closing? Farnesol as Membrane “Liquidizer–Plasticizer”?

A prominent feature of Golgi membranes is their high flexibility and capacity of self-closing. In active Golgi systems, vesicles that are pinched off from the RER will fuse with membranes of the Golgi at its *cis* side. At the *trans* side, the opposite happens. The *trans* vesicles will, in their turn, fuse with the plasma membrane and discharge their content into the cell’s exterior. All these fusion and pinching off require instant closing of the membranes involved; otherwise, the electrical properties of the secreting cells would get severely impaired by the loss of inorganic ions, which are gradient-wise distributed over the membranes. By their nature, sesquiterpenoids are highly flexible molecules as indicated by their high rotatable bond count (7 according to PubChem) and horseshoe-shape form. In addition, they can be anchored in the lipid bilayer part of membranes as shown for the membrane attachment of Ras by Schafer et al. (1989). Already in Barber et al. (1981) reported that JH and two JH analogs can modulate the physical properties of phospholipid bilayers. This raised the prospect that some of their physiological effects may be edited through their influence on the molecular organization of the membrane lipids in particular. Such effect, e.g., on viscosity, for naturally occurring polyisoprenoid alcohols (including farnesol/FLS) was further documented by Murgolo et al. (1989), who determined the horseshoe shape of such compounds as well as their high rotatable bond count, two properties of farnesol/JHs that have only recently been mentioned as being functionally important in the literature on insect endocrinology (De Loof and Schoofs, 2019c, e). Murgolo et al. (1989) (also) suggested a role in protein glycosylation, another important activity in Golgi systems. Thus, in our opinion, farnesol/FLS in the Golgi may serve the role of membrane “liquidizer–plasticizer.” Maybe they can be viewed as the biological counterparts of the phthalates that are used as plasticizers in the polyvinylchloride (PVC) plastic industry. Farnesol/FLS may also have a role in protein glycosylation in the Golgi.

In a former paper, De Loof and Schoofs (2019c) proposed a model for another, transcription-independent, role of farnesol, through the mechanism of prenylation,” for getting inserted in between some of the helices of G protein-coupled receptors. It involves the high flexibility of farnesol/FLS. The self-closing issue of membranes is of course also essential during mitosis, a process that requires Golgi activity (Smith and Malkowski, 2019).

### Why Are Multiple Sites of Farnesol/Farnesol-Like Sesquiterpenoids Synthesis in the Body Required?

The bigger an animal, the higher its membrane turnover. In insects, the CA in larvae may produce enough JH to lubricate the plasma membranes of all cells. This may be facilitated by the fact that the farnesol esters with JH activity (which is orders of magnitude than the biological activity of farnesol itself) ensure that only very low amounts of JHs are needed to accommodate the whole body.

A problem arises when the CA stop secreting JH while internally some tissues continue a function that requires a high membrane turnover. This is the case with the MAGs of *H. cecropia*. But even in the adult stage, when the CA are active, an additional supply of farnesol may be required, e.g., in the gonads with their numerous microvilli and secretory vesicles in some cell types in both males and females (Roth and Porter, 1964; Borovsky et al., 1994a, b).

Our hypothesis is that in insects, CA farnesol secretion suffices of accommodating the needs for plasma membrane flexibility for endocytosis and exocytosis, e.g., in immune reactions. For intensive protein synthesis and release through the RER–Golgi system, additional sites of farnesol/FLS synthesis seem to be required.

### Farnesol/Farnesol-Like Sesquiterpenoids: The Most Ubiquitous Animal Hormone(s)?

Yes, that is the unavoidable conclusion from this review paper. Why was this never proposed before? First, for historical reasons. The very first identification of farnesol was not in animals but in extracts of flowers to be used in perfumes. Its physiological role in plants has not yet been thoroughly investigated. Similar functions as in animal cells (this paper) are likely. Flowers are structures in which massive cell division takes place, an activity for which plasma membrane flexibility and turnover is, by definition, a major issue. Second, a function as “membrane liquidizer” or “membrane lubricant” is the last thing most researchers would think about. Yet, as we know from machine functioning, moving parts need to be lubricated. But this flexibility/lubricant problem is not restricted to animal cells only, but to all eukaryotic ones. That may be an explanation for the omnipresence of farnesol/FLS synthesis by the ubiquitous mevalonate biosynthetic pathway. Third, it is only recently that the key role of the mevalonate biosynthetic pathway in Ca^2+^ homeostasis is put into the picture (De Loof and Schoofs, 2019e).

### Farnesol: Also Part of the Animal’s Pheromone System Like in Plants?

For a long time, farnesol was best known as a component of flower extracts to be used in the perfume industry. Its basic physiological role in plants is still poorly documented. In animal physiology, a possible role as a component of the “smell” of (terrestrial) animals has not yet been considered. In our opinion, there is no reason to assume that with respect to “the (physiologically secondary) perfume aspect,” the situation in animals should differ very much from that in plants.

## Perspectives for Future Research

Only a few possible topics are listed:

### In Vertebrates

The key question: Is farnesol/FLS a hormone in vertebrates? Which stereoisomers do occur? Do SERCAs and SPCA pumps need a farnesol/FLS-like agonist? How would such agonists be attached: by hydrophobic interaction or by prenylation or still in another way? Do all tissues with active Golgi synthesize farnesol/FLS? Does the “inbrome model” of transport also apply to hydrophobic sex steroids?

### In Insects and Other Protostomes

Identify the protein or peptide secreted by the Golgi present in the CA. Are enzymes involved in JH synthesis anchored in the membranes of the Golgi? Why does “exocrine JH” not diffuse into the hemolymph compartment? Does farnesol/FLS, due to its hydrophobic nature, ever end up in the nucleus? How is exocrine JH complementary to endocrine JH?

## Discussion

Farnesol was first detected in floral extracts, which are ingredients for the perfume industry. The name “farnesol” is derived from *Vachellia farnesiana* that had been planted in the garden of Odoardo Farnese in Rome. To our knowledge, the exact physiological role of farnesol in plants is still unknown to date. But such ignorance about the functionality of farnesol/FLS also covers the great majority of all eukaryotes. They all synthesize farnesol. Even in vertebrates, early 2019 farnesol was named “a noble unknown” (De Loof and Schoofs, 2019e). It was thought that its only function was to act as one of the intermediates of the well-documented mevalonate–cholesterol biosynthetic pathway, without having a function by itself in cell physiology. One of the reasons for this way of thinking was that farnesol is not known as a hormone or inbrome or any other type of signaling molecule in vertebrates. Neither are vertebrates known to have a stage in their development in which the body is totally devoid of farnesol/FLS. Finally, neither has it as yet been investigated whether the sesquiterpenoid lactone thapsigargin, upon binding to SERCA or Ca^2+^/Mn^2+^ calcium transport proteins, displaces a putative endogenous possibly FLS ligand.

This contrasts sharply with the situation in insects. They do have such a stage in their development. At the onset the last larval instar of holometabolous insects, the only gland that synthesizes farnesol/FLS (the CA) gets fully inactivated with a drop of farnesol/FLS titers to zero as a result. The hormone secreted by the CA that, when absent, induced metamorphosis had been named “juvenile hormone.” Röller et al. (1967) and Röller and Dahm (1968) identified it as an ester of farnesol. Farnesol itself has biological activity in typical bioassays for detecting JH activity (for a picture of a positive reaction, see De Loof et al., 2014, open access). Its activity is weak compared to the much higher activity of its esters.

In rodents and man, farnesol, which is biosynthesized in the brain and other tissues, acts as a potent blocker of N-type voltage-gated Ca^2+^ channels (Roullet et al., 1999) and Luft et al. (1999). This discovery causally linked farnesol to the control of Ca^2+^ homeostasis. However, for one reason or another, these data did not draw the attention of the vertebrate endocrine community, but they did, with some delay, of insect endocrinologists who searched for the nature of the membrane receptor(s) of JH in insects (De Loof and Schoofs, 2019b). That is the reason why in this review paper, the majority of data and models come from insect endocrinology: data in vertebrates are almost non-existent.

The fact that all eukaryotes, both uni- and multicellular ones, produce farnesol is compatible with a key role in Ca^2+^ homeostasis. Indeed, Ca^2+^ is the most ubiquitous toxin on earth for all eukaryotes; they all have to counter the influx of excess Ca^2+^ from the extracellular environment of cells.

The idea of a functional link between the Golgi and farnesol/FLS emerged from a serendipitous reexamination (De Loof and Schoofs, 2019d) of a paper by Schmialek (1961). It triggered the idea that the most logical explanation for the fact that farnesol is found in substantial concentrations in excrements of the mealworm *Tenebrio* was that farnesol must be unidirectionally transported from the gut’s epithelial cells into the gut’s lumen. The only cell organelle that can enable this is the RER–Golgi system. Paroulek and Sláma (2014) showed for the first time that JH I that turned out to be synthesized by the MAGs themselves of the silk moth *H. cecropia*, and not in their CA as had been assumed before, is *unidirectionally* transported, not into the hemolymph, but into the genital system of the female during copulation. This is a strange way of transport of a hydrophobic hormone that had been assumed to freely, not unidirectionally, diffuse in cells. It raised the question: Is, perhaps, the Golgi system involved in the production and transport of all cells with an active Golgi?

Upon reexamining the older literature, it became apparent that the presence of active Golgi in the CA was already well documented since 1964 (Scharrer, 1964; Bradley and Edwards, 1979), but these data got buried under the massive number of novel developments in the endocrine field. Yet, the presence of active, protein-secreting Golgi in the CA deserves to be reanalyzed. It could, perhaps, yield a very potent growth factor, the importance of which might exceed that of JH, which may rather be an adjuvant than a growth factor by itself, an idea already formulated by Sláma (2013). Our knowledge of Ca^2+^ homeostasis in the CA is limited (Allen et al., 1992a, b; Granger et al., 1995) and needs further exploration.

Farnesol and its esters are not at all the only hydrophobic hormones present in animals. Some sex steroids, e.g., testosterone and estradiol are also poorly soluble in water (see PubChem). It looks plausible to assume that the major mechanisms of their transport and mode of action at the level of the plasma membrane may be similar to those of farnesol/JHs.

The conclusions of this paper are based on a combination of experimental data and logical deduction. We fully agree with the objection that, for the moment being, too few experimental data are available to fully generalize “insect data” to ubiquitous mechanisms that we suppose operate in all eukaryotes. We nevertheless maintain our generalizations because we assume that the Golgi system has been well conserved in evolution for billions of years, along with the mevalonate biosynthetic pathway that is also omnipresent in eukaryotes because both serve an essential function, namely, in Ca^2+^ homeostasis. Otherwise, they would have disappeared in some subdivisions of the eukaryotes, but they did not.

In insect endocrinology, the idea that the CA is the unique site of synthesis of JH(s) was vigorously “defended” for over two decades against disagreeing researchers: no way to publish “aberrant” experimental data. Today’s situation is very different. If the two major functions of the Golgi, namely, roles in Ca^2+^ homeostasis and in farnesol/FLS production, are accepted, the novel paradigm says that it is normal and almost self-evident that all protein-secreting cells and tissues may produce their own FLSs. Whether there are quantitative and qualitative (types of isomers) differences remains to be investigated. The data that farnesyldiphosphate synthase is only abundant in the CA and not in any other tissue known to synthesize farnesol/FLS (Castillo-Gracia and Couillaud, 1999) suggest that the CA are indeed the master gland that influences all cells of the body. Apparently, some (intra)cellular functions, in particular in the case of active protein secretion by the RER–Golgi, the massive Ca^2+^ removal and membrane turnover in the Golgi require an additional local site of synthesis.

With this review, we intend to stimulate research in both basic and medical sciences into the full role of the Golgi. Malfunctioning of the Golgi system is implicated in several diseases, e.g., breast cancer and Haily–Haily disease (Chen et al., 2019). De Loof and Schoofs (2019a) also suggested that a dysfunctional mevalonate pathway may disturb Golgicrine activity (as it does in insects) and be a major inducer of Alzheimer’s disease.

We also intend to contribute to eliminate the goal-oriented thinking on evolution, which is still unconsciously propagated in textbooks: see the “milk production case” in this paper in which many people still mistake result for physiological necessity.

## Author Contributions

Both authors jointly conceived and wrote the manuscript.

## Conflict of Interest

The authors declare that the research was conducted in the absence of any commercial or financial relationships that could be construed as a potential conflict of interest.
